# Surfactant Uptake Dynamics in Mammalian Cells Elucidated with Quantitative Coherent Anti-Stokes Raman Scattering Microspectroscopy

**DOI:** 10.1371/journal.pone.0093401

**Published:** 2014-04-07

**Authors:** Masanari Okuno, Hideaki Kano, Kenkichi Fujii, Kotatsu Bito, Satoru Naito, Philippe Leproux, Vincent Couderc, Hiro-o Hamaguchi

**Affiliations:** 1 Department of Chemistry, School of Science, The University of Tokyo, Bunkyo-ku, Tokyo, Japan; 2 Safety Science Research Laboratories, Kao Corporation, Haga-Gun, Tochigi, Japan; 3 Analytical Science Research Laboratories, Kao Corporation, Haga-Gun, Tochigi, Japan; 4 Institut de Recherche XLIM, UMR CNRS, Limoges, France; 5 Institute of Molecular Science and Department of Applied Chemistry, National Chiao Tung University, Hsinchu, Taiwan; Biological Research Centre of the Hungarian Academy of Sciences, Hungary

## Abstract

The mechanism of surfactant-induced cell lysis has been studied with quantitative coherent anti-Stokes Raman scattering (CARS) microspectroscopy. The dynamics of surfactant molecules as well as intracellular biomolecules in living Chinese Hamster Lung (CHL) cells has been examined for a low surfactant concentration (0.01 w%). By using an isotope labeled surfactant having CD bonds, surfactant uptake dynamics in living cells has been traced in detail. The simultaneous CARS imaging of the cell itself and the internalized surfactant has shown that the surfactant molecules is first accumulated inside a CHL cell followed by a sudden leak of cytosolic components such as proteins to the outside of the cell. This finding indicates that surfactant uptake occurs prior to the cell lysis, contrary to what has been believed: surface adsorption of surfactant molecules has been thought to occur first with subsequent disruption of cell membranes. Quantitative CARS microspectroscopy enables us to determine the molecular concentration of the surfactant molecules accumulated in a cell. We have also investigated the effect of a drug, nocodazole, on the surfactant uptake dynamics. As a result of the inhibition of tubulin polymerization by nocodazole, the surfactant uptake rate is significantly lowered. This fact suggests that intracellular membrane trafficking contributes to the surfactant uptake mechanism.

## Introduction

Interactions of surfactants with living cells are of considerable interest with regard to their biological functions including cellular toxicity [1]. Understanding their toxicological mode of action is highly important in order to assess and control their safety on human exposure [2]–[4]. Previous studies have shown that microorganisms solubilization by surfactants occurs with cell lysis, in which the cell membrane is degraded by surfactants with eventual breakdown of the whole cell [5]–[8]. However, the dynamical process of surfactant action in single living cells is still unexplored because of the lack of the mean to visualize surfactant molecules *in vivo* and *in situ*. In the present study, we use a recently-emerging new tool, CARS microspectroscopy [9]–[13], which is powerful for studying lipid molecules in living cells. We also use an isotope labeled surfactant (d_25_-sodium dodecyl sulfate (SDS)) and visualize the dynamics of surfactant molecules in the cell lysis process. Deuterium substitution enables us to selectively trace the SDS molecules among a number of unlabeled biomolecules [10], [14]–[16]. d_25_-SDS gives CD stretch bands in the 2000–2200 cm^−1^ spectral region, which is a “window” of Raman spectra of unlabeled biomolecules, facilitating its selective detection.

Although fluorescence labeling is a powerful technique for tracing the dynamics of lipid molecules in a living cell [17]–[19], introduction of fluorophores may well perturb the physical and chemical properties of the surfactant, such as charge, hydrophobicity, and hydrophilicity. Isotope labeling in vibrational spectroscopy is well established as a unique method for distinguishing the labeled molecule from the others. A great advantage of isotope substitution is the same chemical properties between the labeled and unlabeled species. Recently, we have developed quantitative CARS microspectroscopy [20], which combines multiplex CARS microspectroscopy with the maximum entropy method (MEM) [21]–[23]. The spectral coverage in this method is broad enough (>3000 cm^−1^) to observe all the fundamental vibrational modes including not only the C-H, C-D stretch regions but also the fingerprint region. Thus, quantitative CARS microspectroscopy with deuterium substitution is ideally suited for real-time spectral tracing of cells and the surfactant molecules during the lysis process.

## Materials and Methods

### Quantitative CARS microspectroscopy

We use a CARS microspectrometer developed in our laboratory. The details of the CARS system are described in File S1 [20].

### Sample

Chinese Hamster Lung (CHL) cells [24], which are routinely used for toxic evaluation, were used as a sample in the present study. CHL cells were incubated at 37°C under 5% CO_2_. The culture medium were D-MEM (Dulbecco's modified essential medium, Gibco) supplemented with 10% fetal bovine serum (FBS).

### Chemicals

^2^H-substituted sodium dodecyl sulfate (d_25_-SDS) was used as a surfactant. The culturing media was suspended with d_25_-SDS solution (0.1 wt% SDS in PBS buffer) so that the final concentration of d_25_-SDS was approximately 0.01 w%, 0.3 mM. This concentration is too low to be detected by the CARS microspectroscopic system. We found no spectroscopic signature of the CD stretch from the suspended medium. Nocodazole was used as an inhibitor of intracellular membrane trafficking in CHL cells [19]. It inhibits the polymerization of tubulin and subsequent formation of microtubes. Since nocodazole is not soluble in water, it was solved in dimethylsulfoxide. This solution was added to the medium with the final nocodazole concentration of 25 µM. Cells were incubated for 30 min after the addition of nocodazole.

## Results and Discussion

### Cell lysis efficiency

We first analyzed the cell lysis efficiency of d_25_- SDS as a model surfactant. Cultured CHL cells were scrape-harvested to micro-centrifuge tubes and spin-downed. Then, the supermatants were removed from the solution. Cell pellets were then resuspended to SDS solutions at each concentration of 1, 0.1, 0.01, 0.001 and 0.0001 w% by voltex for 1 min. The suspensions were spin-downed and we checked the degree of cell lysis. High concentration of 1∼0.1 w% of SDS solution apparently lyses the CHL cells. On the other hand, below 0.001 w% of SDS solution, the cells remain stable as a pellet (Table 1). These results suggest that the ‘0.01 w% concentration of SDS solution’ is approximately a threshold of CHL cell lysis and denaturation. Thus, we determined the concentration of SDS (0.01 w%, 0.3 mM) for tracing the lysis process of CHL cells with CARS microspectroscopy.

**Table 1:** The concentration dependence of d_25_-SDS cell lysis efficiency; ++ corresponds to clear cell lysis, +: moderate cell lysis, −: remaining stable.

SDS concentration [w%]	1	0.1	0.01	0.001	0.0001
CHL lysis efficiency	++	++	+	−	−

### CARS measurement of a living CHL cell without any treatment

**Figure 1:**
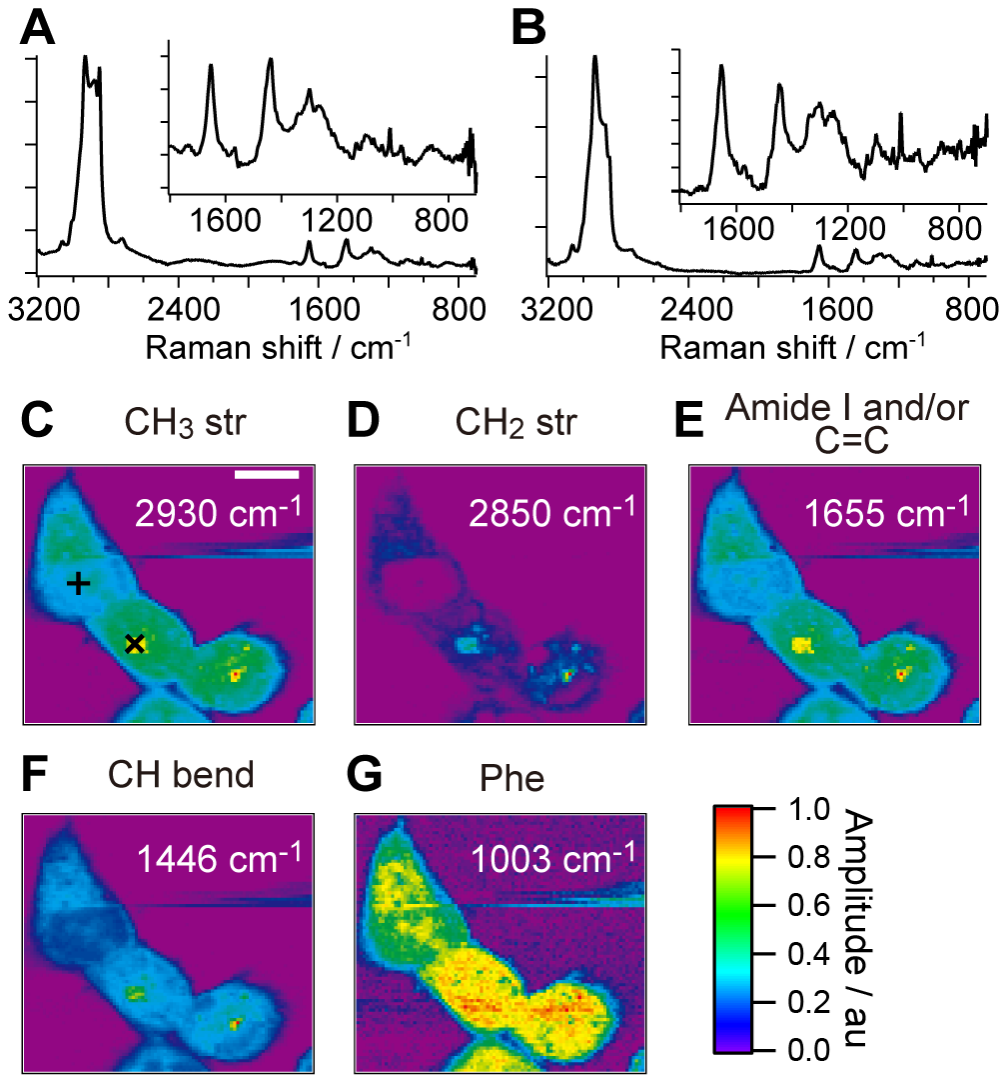
Im[χ^(3)^] spectra and images from a CHL cell. Im[χ^(3)^] spectra from the two points of the CHL cell. **A** and **B** are obtained from the points indicated as × and + in **C**, respectively. The inset of each spectrum is the expanded spectrum in the fingerprint region. The exposure time is 50 msec. Im[χ^(3)^] images at 2930 cm^−1^ (**C**), 2850 cm^−1^ (**D**), 2655 cm^−1^ (**E**), 2446 cm^−1^ (**F**) and 1003 cm^−1^ (**G**), respectively. The scale bar in the image is 10 µm. The image consists of 91×81 pixels and the exposure time for each pixel is 50 msec. Each image is normalized at the intensity maximal of each band.

Figures 1A and 1B show typical CARS spectra (Im[χ^(3)^]) obtained from two different points in a CHL cell. Using the MEM and a singular value decomposition analysis [25], we obtain Im[χ^(3)^] spectra with a high signal-to-noise ratio (∼10^3^ in the CH stretching region and ∼10^2^ in the fingerprint region) from observed multiplex CARS spectra. Compared with the reported Raman spectra of known biomolecules [26], it is clear that Figures 1A and 1B show characteristic spectral features of lipids and proteins, respectively. Based on the Im[χ^(3)^] spectra obtained from a cell, we construct Im[χ^(3)^] images, whose amplitude is proportional to the molecular concentration. Figures 1C–H show the Im[χ^(3)^] images of CHL cells at 2930 cm^−1^, 2850 cm^−1^, 1655 cm^−1^ 1446 cm^−1^ and 1003 cm^−1^. They are assigned to the CH_3_ stretch, the CH_2_ stretch, the superposition of the C = C stretch of lipid chains and the amide I of proteins, the CH bend and the phenylalanine residues, respectively. The image of the CH_2_ stretch mode shows localized and intense signals inside the cell. This image mainly reflects the distribution of lipid molecules such as lipid droplets. On the other hand, the image of phenylalanine is homogeneous in the cell, reflecting the homogeneous distribution of proteins. The images of CH_3_ stretch, C = C and/or amide I and CH bend modes seem to be the sum of the former two groups. The relatively strong phenylalanine signal and no CH_2_ stretch signal are observed around the center of the cells. These areas correspond to the nuclei of the cells. It has been confirmed that the CARS images do not show any appreciable change for an hour, which indicates that the laser irradiation causes no serious photo or thermal damage to the CHL cells on this time scale (the time-resolved CARS images are shown in Figure S1 in File S1).

### CARS measurement of a living CHL cell with surfactant

**Figure 2:**
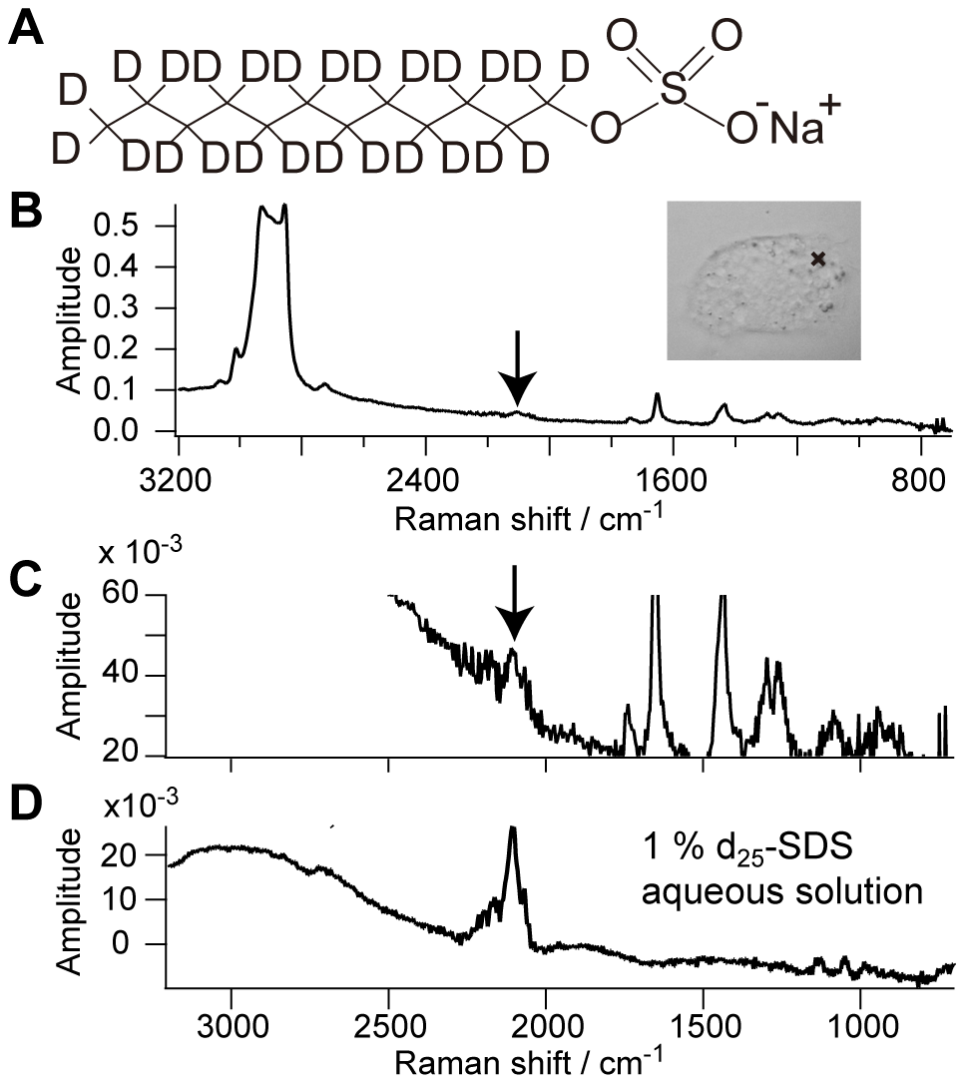
SDS molecules are condensed in a CHL cell. **A**. The molecular structure of d_25_-SDS. **B**. Im[χ^(3)^] spectrum obtained from one point of a CHL cell indicated as the cross in the inset several minutes after the addition of d_25_-SDS. **C**. The expanded spectrum of **B**. **D**. Im[χ^(3)^] spectrum of 1% d_25_- SDS aqueous solution. The exposure time for **B**–**D** is 50 msec and **B**–**D** are measured under the same experimental condition.

First, we focus on a CHL cell with the addition of the surfactant SDS (Fig. 2A). Figures 2B and 2C are Im[χ^(3)^] spectra obtained from one particular position (indicated in the inset of Fig. 2B) of the CHL cell with the addition of d_25_-SDS. Surprisingly, the CD stretch band at 2100 cm^−1^ is found in the cell only in several minutes after the addition of d_25_-SDS. The concentration of d_25_-SDS inside the cell is estimated to be approximately ∼10 mM by comparing the signal intensity with that of 1 w% (32 mM) d_25_-SDS aqueous solution in Fig. 2C. This relatively high concentration indicates that the observed CD stretch intensity does not originate from cell membranes, which are too thin to accumulate a large number of CD carrying molecules to make a high concentration within the laser focal volume, but is more likely to come from the bulk cytoplasm of the cell. Then, the SDS molecules must be taken into the cell efficiently through cell membranes. The concentration of 10 mM is 30 times higher than the original concentration of d_25_-SDS in the medium, namely, 0.3 mM. It should be noted, however, that the observed CD stretch band may not originate solely from d_25_-SDS. Living cells can modify the molecular structure of the surfactant through metabolism, while these molecules are unlikely to be completely metabolized in this time range [27]. We need to confirm that what we observe is not the surfactant molecule itself but the CD stretch Raman signal.

**Figure 3:**
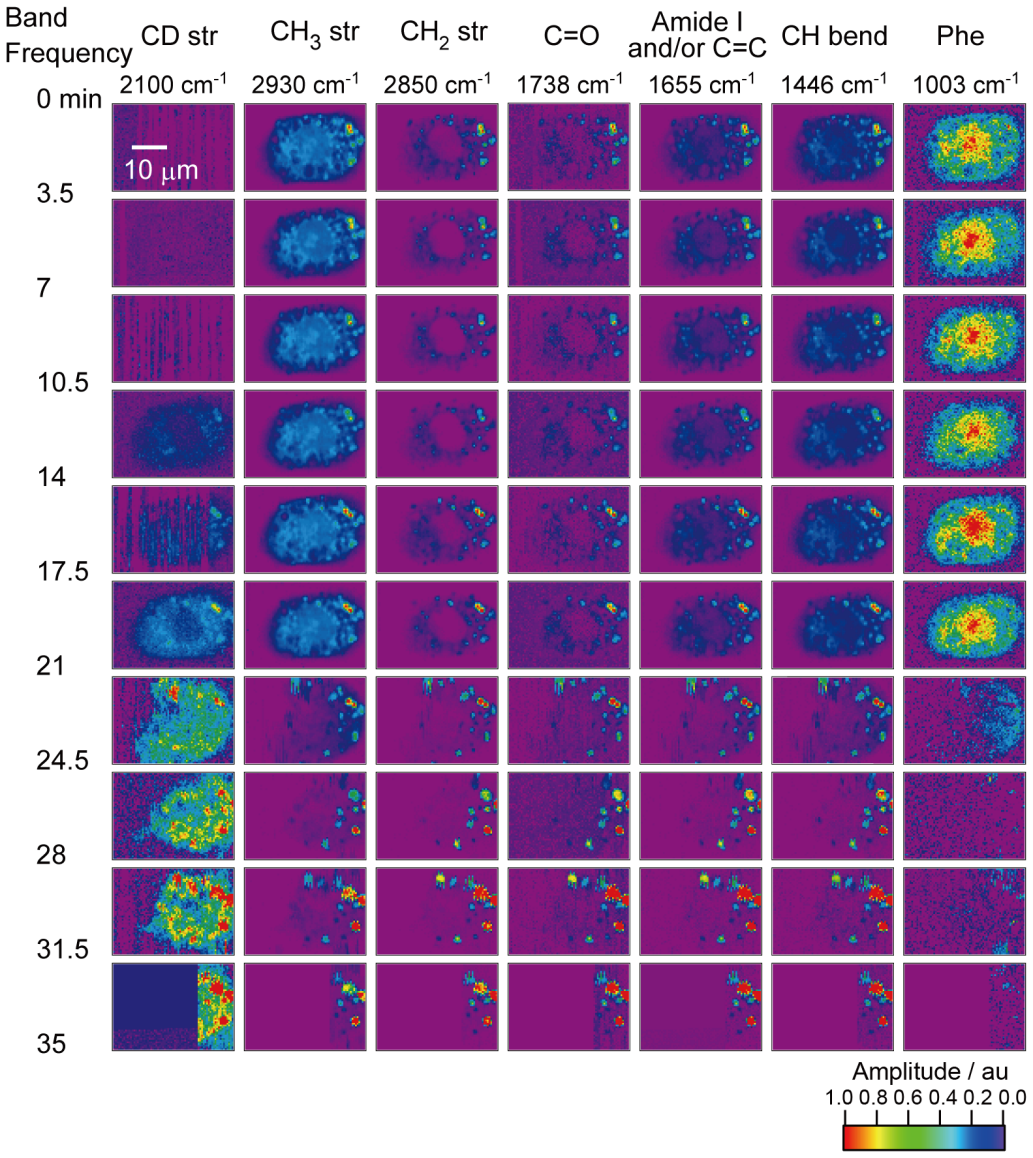
Time-resolved Im[χ^(3)^] images of the CHL cell with the surfactant. The scale bar in the image is 10 µm. The image consists of 71×51 pixels and the exposure time for each pixel is 50 msec. Each row of the CARS images is measured every 3.5 min. Each column is normalized at the intensity maximal of each band.

Next, the surfactant uptake dynamics is traced in the time-resolved CARS mapping study. Figure 3 shows the time-resolved CARS mapping images of a d_25_-SDS treated CHL cell over a time span of 35 min. The CARS measurement of the CHL cell was started immediately after the addition of d_25_-SDS.

The dynamics of the surfactant uptake and cell lysis is clearly visualized in this experiment. The CD stretch signal increases with time from 0 to 21 min. The signal intensities of all the bands, in particular that of the 1003 cm^−1^ band, drastically change around 23 min. The Raman band of phenylalanine residues almost disappears at 24.5 min, while the strong Raman signal of lipid is still observed between 24.5 to 35 min. The image of the CD stretch is similar to that of the CH bend mode, and is totally different from that of phenylalanine residues (proteins). Taking into account of the assignment of these bands as discussed above, this fact suggests that the surfactant molecules are widely spread over the cell cytoplasm. There are several possible pathways of surfactant transport through cellular membranes; protein-mediated transport, flip-flop, diffusion, and endocytosis. Protein-mediated transportation such as ion channels and transporters plays an important role to transport molecules through cellular membranes. Since SDS is relatively large and amphipathic, it should not be transported through ion channels. Furthermore, no transporter for SDS has been known. Protein-mediated transportation is thus unlikely taking place in the present system. Second, trans-membrane lipid translocation (so called flip-flop) could introduce SDS into intracellular membrane. However, the flip-flop mechanism cannot explain accumulation of SDS in cytoplasm. Third, the diffusion process could contribute to the SDS accumulation in the cytoplasm, but it will be shown in the next subsection that it is not the main pathway of the SDS accumulation. Therefore, we exclude three possibilities, protein-mediated transport, flip-flop, and diffusion in the surfactant uptake process.

Based on the above discussion and our CARS results, the overall dynamic behavior inside the cell is summarized in the following way. First, the surfactant molecules are diffused in the medium, and inserted into the cell membranes. Then, they are intenalized into the cell possibly through the endocytosis [28], [29]. In the present study, it was difficult to identify each endosome in cytoplasm through endocytosis. Taking into account of the final concentration of the surfactant inside the cell (∼10 mM), it is highly likely that the cell actively uptakes the surfactant into itself.

The SDS molecules inserted in the endosome membrane are ongoingly accumulated, and condensed inside the cell in ∼20 min. Judging from the CARS images in 20 min, the strong CD stretch signal is observed from the points where the CH_2_ stretch signal is strong, indicating that the SDS molecules are accumulated in the lipid droplets. It is an intriguing clue to the elucidation of lipotoxicity [30]. Finally, the morphology of the cell drastically changes due to the disruption of the cell membranes, indicating that the surfactant lyses the cell after all. The time-resolved images of phenylalanine residues (proteins) suggest that the concentration of proteins is dramatically lowered at 24.5 min due to the leak of proteins from the cell. Some part of the cell, most probably reflecting the lipid droplets, still remains at the same positions as cell debris even after the disruption of cell membranes.

**Figure 4:**
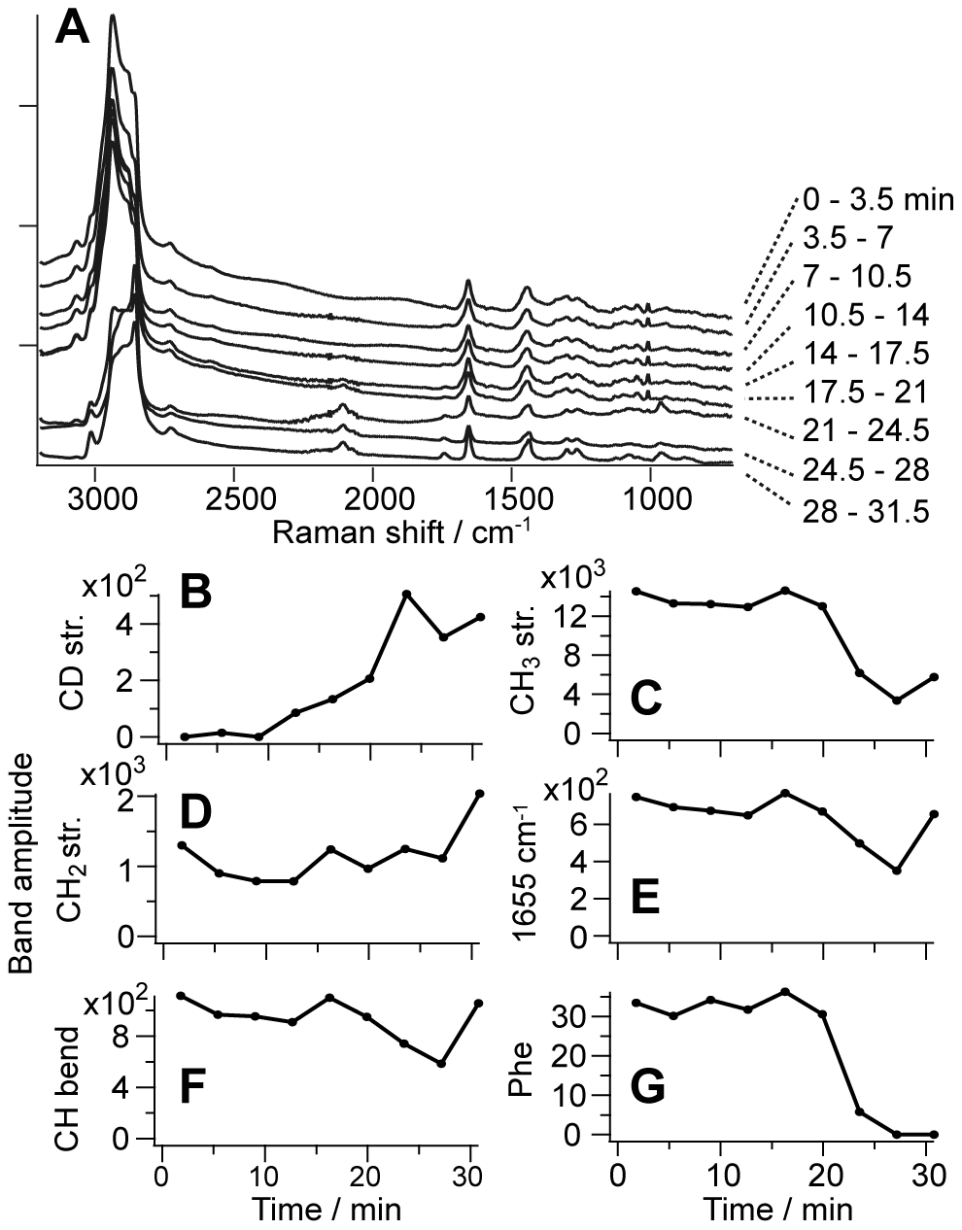
Accumulation of SDS in a CHL cell and subsequent cellular death. **A**. Time-resolved Im[χ^(3)^] spectra obtained with the summation over all the spectra in the cell shown in Fig. 3. Time-profiles of band amplitudes at 2100 cm^−1^ (**B**), 2930 cm^−1^ (**C**), 2850 cm^−1^ (**D**), 1655 cm^−1^ (**E**), 1446 cm^−1^ (**F**) and 1003 cm^−1^ (**G**).

For the purpose of quantitative analysis, we sum up the spectral profiles of the Im[χ^(3)^] obtained in the whole cell at each time. Figure 4A shows the time-resolved Im[χ^(3)^] spectra from the whole cell. The CD stretch signal at 2100 cm^−1^ gradually increases after the addition of the surfactant. The decreases of the CH_3_ stretch at 2930 cm^−1^ and phenylalanine residues at 1003 cm^−1^ at 21 min indicate the leak of proteins from the cell. We fitted each Im[χ^(3)^] spectrum by the sum of Lorentzian functions shown in Fig. 4A, and obtain the time profiles of the 2100 cm^−1^, 2930 cm^−1^, 2850 cm^−1^, 1655 cm^−1^, 1446 cm^−1^ and 1003 cm^−1^ Raman bands as shown in Fig. 4B–G. In the CH stretching region, we fitted the spectra with the sum of three Lorentzian functions whose band positions are 2930, 2880 and 2850 cm^−1^. The band amplitude is plotted against the time delay from the addition of the surfactant in each time-profile. Figure 4B strongly suggests that the surfactant is gradually taken into the cell and Fig. 4G indicates that the proteins leak suddenly from the cell. On the other hand, the CH_2_ signal intensity does not change with time (Fig. 4D). This indicates that the total amount of lipid molecules remains almost the same in the region of interest during the measurement. The sharp rise of the (2100 cm^−1^,) 2930 cm^−1^, 2850 cm^−1^, 1655 cm^−1^ and 1446 cm^−1^ around 30 min can be ascribed to the laser trapping effect of lipid droplets.

In order to confirm the reproducibility of the observed phenomenon we have measured nine cells. The time-resolved profiles of the 1003 cm^−1^ and 2100 cm^−1^ Raman bands of the nine cells are summarized in Figure S2 in File S1. Even though they show different temporal profiles, it is certain that the CD stretch signal increases gradually and the phenylalanine signal decreases suddenly (15∼30 min). This suggests that cells are lysed by the surfactant on the similar time scale through the same mechanism. Interestingly, cell lysis is initiated after the 2100 cm^−1^/1003 cm^−1^ amplitude ratio (CD stretch/Phe) reaches a certain level (3 to 10). Although it is difficult to discuss this finding quantitatively, because the sizes and conditions of the cells are different from one another, there seems to exist a threshold of SDS concentration for cell lysis to occur. The 2100 cm^−1^/1003 cm^−1^ amplitude ratio could thus be used as a cell-lysis progress indicator.

### CARS measurement of a living CHL cell with surfactant and inhibitor

The time-resolved CARS measurements shown in Figs. 3 and 4 indicate that CHL cells actively internalize the surfactant molecules. Next, we investigate the effect of an inhibitor of intracellular membrane trafficking, nocodazole, upon the surfactant uptake. Nocodazole is known as an inhibitor of tubulin polymerization and the subsequent formation of microtubes [31], [32].

**Figure 5:**
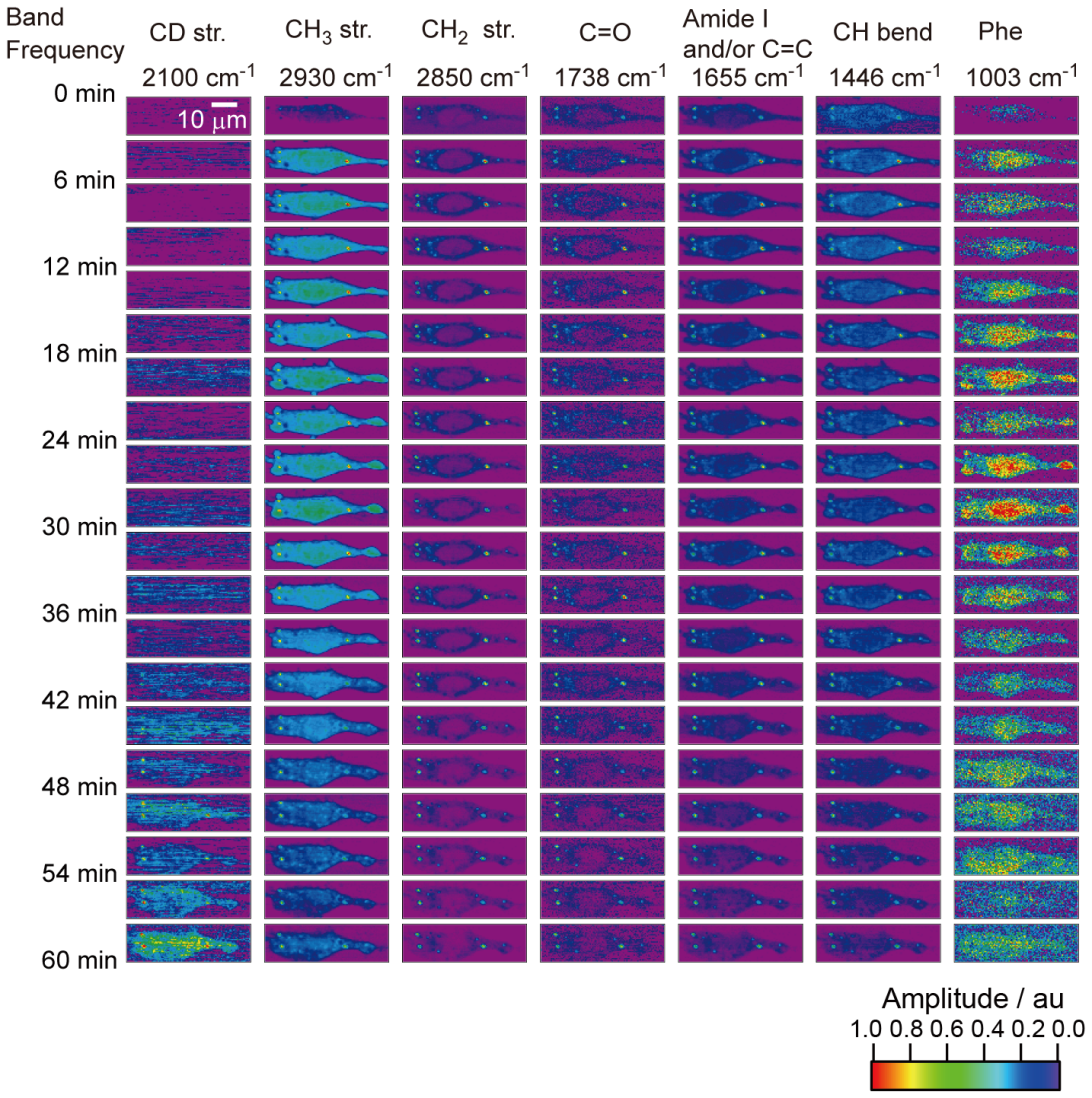
Time-resolved Im[χ^(3)^] images of the CHL cell with the surfactant and inhibitor of intracelluar membrane trafficking. The scale bar in the image is 10 µm. The image consists of 101×31 pixels and the exposure time for each pixel is 50 msec. Each row of the CARS images is measured every 3 min. Each column is normalized at the intensity maximal of each band.

**Figure 6:**
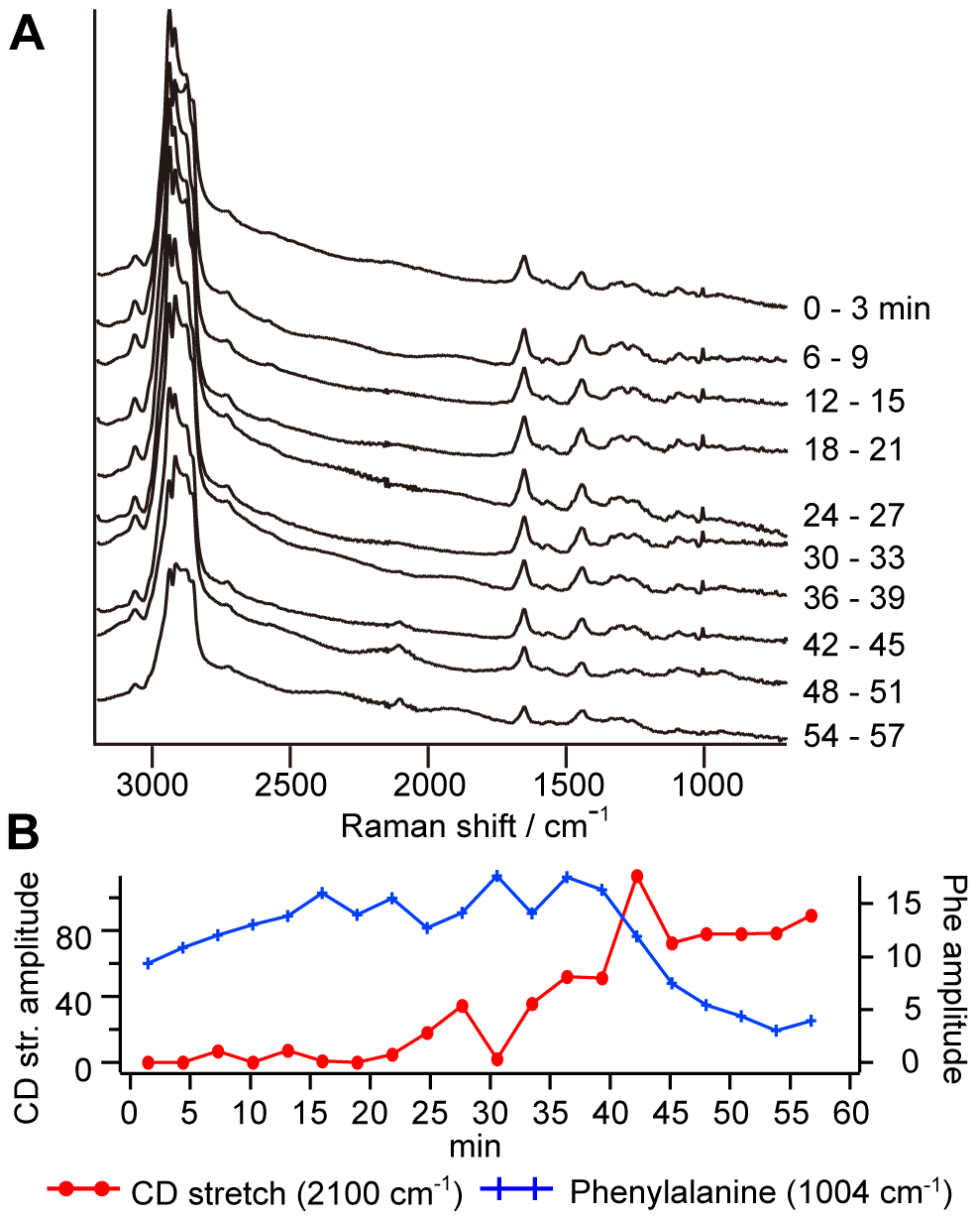
Nocodazole lowers the surfactant uptake rate of a CHL cell. **A**. Time-resolved Im[χ^(3)^] spectra obtained with the summation over all the spectra in the cell shown in Fig. 5. **B**. Time-profiles of band amplitudes at 2100 cm^−1^ (circle, left axis) and 1003 cm^−1^ (cross, right axis).

Figure 5 shows the results of the time-resolved CARS imaging experiment on a nocodazole treated CHL cell (incubated with nocodazole) with d_25_-SDS over a time span of 60 min. Highly localized distributions of lipid bands are clearly observed, while the image of proteins is homogeneously distributed. In addition, the gradual increase of the CD stretch ascribed to the surfactant uptake is also observed. Using the same procedure as the experiment without nocodazole, we sum up all the spectral profiles obtained from the whole cell at each time. Figure 6A shows the time-resolved Im[χ^(3)^] spectra from the whole cell. The strong background signal from dimetylsulfoxide (solvent of nocodazole) is already subtracted from the observed spectra. The spectral profiles in the CH stretch region (2700∼3000 cm^−1^) are slightly distorted by this background subtraction. Figure 6B shows the time-profiles of the 2100 cm^−1^ (the CD stretch mode; circle, left axis) and 1003 cm^−1^ (the phenylalanine residues; cross, right axis) Raman band amplitudes obtained from Fig. 6A. The gradual increase of the CD stretch signal is observed in this case as well as in the experiments without nocodazole. On the other hand, the decrease of the 1003 cm^−1^ band is not as fast as that without nocodazole. Moreover, it takes approximately an hour for the cell to lyse, while several tens of minutes in the experiments without nocodazole. This experimental result indicates that nocodazole, an inhibitor of polymerization of tubulin, slows down the surfactant uptake dynamics. We have measured six cells to check the reproducibility (see Figure S3 in File S1). We ascribe this change to the inhibition of the membrane trafficking, which plays a key role to transport of the surfactant to organelles. Now, we can discuss the main contribution of the surfactant uptake we observed. While the inhibitor does not affect diffusion, it is ensured that nocodazole slows down/stops endocytosis. The fact that nocodazole slows down the rate of the surfactant uptake strongly suggests that the surfactant uptake is controled mainly by endocytosis.

The increase of the CD stretch band amplitude and the decrease of phenylalanine residues seem to take longer time (30∼60 min) compared with the experiments without nocodazole (15∼30 min). The decrease of phenylalanine residues and the increase of the CD stretch band are almost synchronized. The final amount of the surfactant inside the cell is similar for the experiments with and without nocodazole. However, the total amount of phenylalanine inside the cells decreases gradually with nocodazole, while it suddenly occurs without nocodazole due to the cell lysis. This fact indicates that cell lysis with SDS occurs in different ways for CHL cells with and without nocodazole. Nocodazole seems to change the surfactant uptake and cell lysis mechanism profoundly.

## Conclusion

Multiplex CARS microspectroscopy has been used to study the surfactant uptake dynamics in living CHL cells. The dynamics of surfactant accumulation inside the cells and subsequent cell lysis is clearly observed with several-minutes temporal resolution. Given that the observed CD stretch signal exclusively originates from the surfactant, the concentration of the surfactant in a CHL cell is estimated to be several 10 mM. The surfactant is found to accumulate in the overall cell cytoplasm. There seems to be a threshold of the surfactant concentration for cell lysis to occur. By adding nocodazole as an inhibitor of active transport, the surfactant uptake speed significantly decreases. We have thus proved that the surfactant uptake and following cell lysis are closely related with intracellular membrane trafficking in living cells. This result indicates that living cells retain the surfactant not only in the plasma membrane but also in the whole intracellular membrane system and that the intracellular metabolism is related to detoxification of surfactant. The findings in the present study will contribute not only to the fundamental understanding of surfactant/cell interactions but also to the improvement of surfactant toxicity in our daily life.

## Supporting Information

File S1 Experimental setup. Figure S1, Time-resolved Im[χ^(3)^] images of CHL cells without any treatment. Figure S2, Time-profiles of the Im[χ^(3)^] amplitudes of the 2100 cm^−1^ (CD stretch; red circle, left axis) and 1004 cm^−1^ (phenylalanine; blue cross, right axis) obtained from nine CHL cell without the addition of nocodazole. Figure S3, Time-profiles of the Im[χ^(3)^] amplitudes of the 2100 cm^−1^ (CD stretch; red circle, left axis) and 1004 cm^−1^ (phenylalanine; blue cross, right axis) obtained from six CHL cell with the addition of nocodazole.
(DOCX)
