# Supplementary material for: Surfactant Uptake Dynamics in Mammalian Cells Elucidated with Quantitative Coherent Anti-Stokes Raman Scattering Microspectroscopy
Source: PLoS One. 2014 Apr 7;9(4):e93401. doi: 10.1371/journal.pone.0093401 (PMC3977816; doi:10.1371/journal.pone.0093401)
Supplement: File S1 — Experimental setup. Figure S1, Time-resolved Im[χ(3)] images of CHL cells without any treatment. Figure S2, Time-profiles of the Im[χ(3)] amplitudes of the 2100 cm−1 (CD stretch; red circle, left axis) and 1004 cm−1 (phenylalanine; blue cross, right axis) obtained from nine CHL cell without the addition of nocodazole. Figure S3, Time-profiles of the Im[χ(3)] amplitudes of the 2100 cm−1 (CD stretch; red circle, left axis) and 1004 cm−1 (phenylalanine; blue cross, right axis) obtained from six CHL cell with the addition of nocodazole. (DOCX) [file pone.0093401.s001.docx]

**Supporting information**

**Experimental setup**

A dual-output compact laser source has been used. The pump source is a passively Q-switched 1064-nm microchip laser (pulse width: < 1-ns, repetition rate: 33 kHz, average power: ~300 mW). The laser beam is equally divided into two with a beam splitter. One part is directly used for the pump radiation (~5 kW peak power; ~150mW average power; <0.1 cm^-1^ linewidth) of the CARS process after adjusting its power with a variable neutral density filter. The other is introduced into a 6-m-long air-silica photonic crystal fiber, characterized by a 2.5-μm hole diameter and a 4-μm hole-to-hole spacing, which results in a zero-dispersion wavelength of 1040 nm for the fundamental guided mode of the structure. The strong third-order nonlinear effects, that occur all along the propagation in the fiber, create white laser emission at the fiber output, with >100 µW/nm spectral power density from 1.05 μm to 1.6 μm. This white laser beam is used for the Stokes radiation of the CARS process. The pump and Stokes beams pass several interference filters to eliminate anti-Stokes spectral components, are superimposed by a notch filter, and are introduced into the modified inverted microscope (Nikon: ECLIPSE Ti). The pump and Stokes pulses are tightly focused onto the sample with an objective (Nikon: Plan Fluor 100x/NA 1.3). The CARS signal generated by the sample is collected by another objective (Nikon: Apo NIR 60x/NA 1.0) and guided into a spectrometer (Princeton Instruments: SpectraPro2300i and PIXIS 100BR). The laser power of both the pump and Stokes lasers was 10 mW.


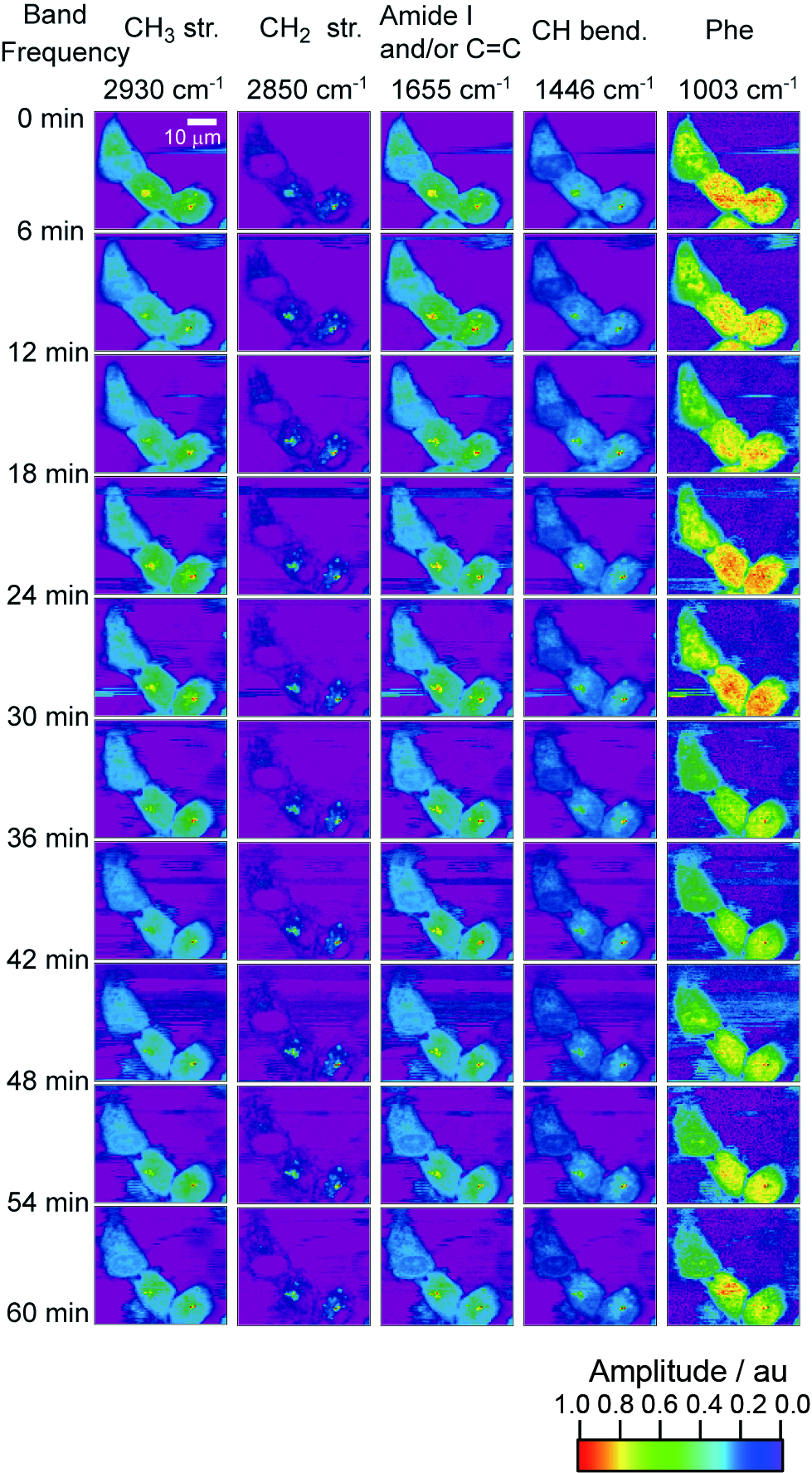


Figure S1 Time-resolved Im[χ^(3)^] images of CHL cells without any treatment


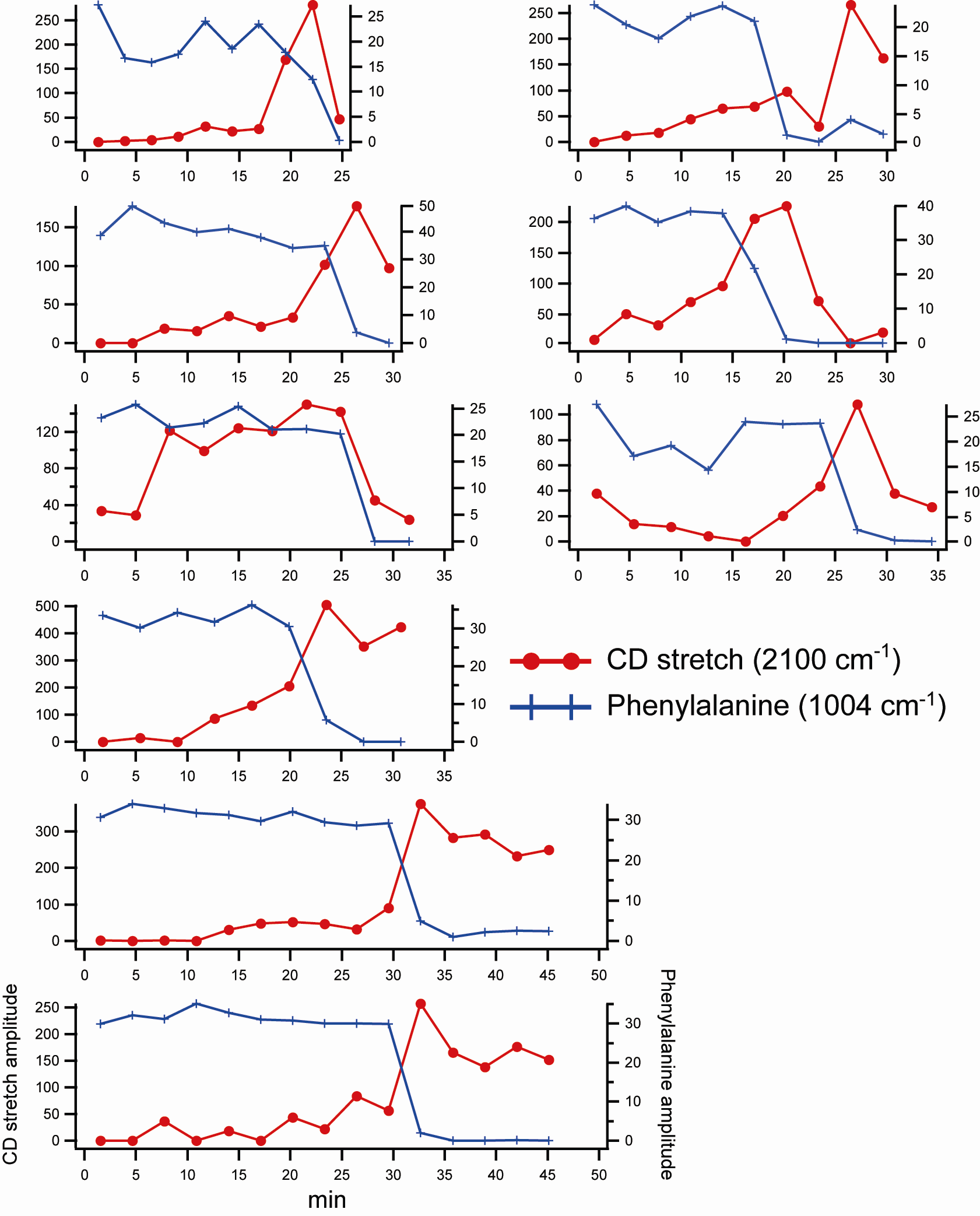


Figure S2 Time-profiles of the Im[χ^(3)^] amplitudes of the 2100 cm^-1^ (CD stretch; red circle, left axis) and 1004 cm^-1^ (phenylalanine; blue cross, right axis) obtained from nine CHL cell without the addition of nocodazole.


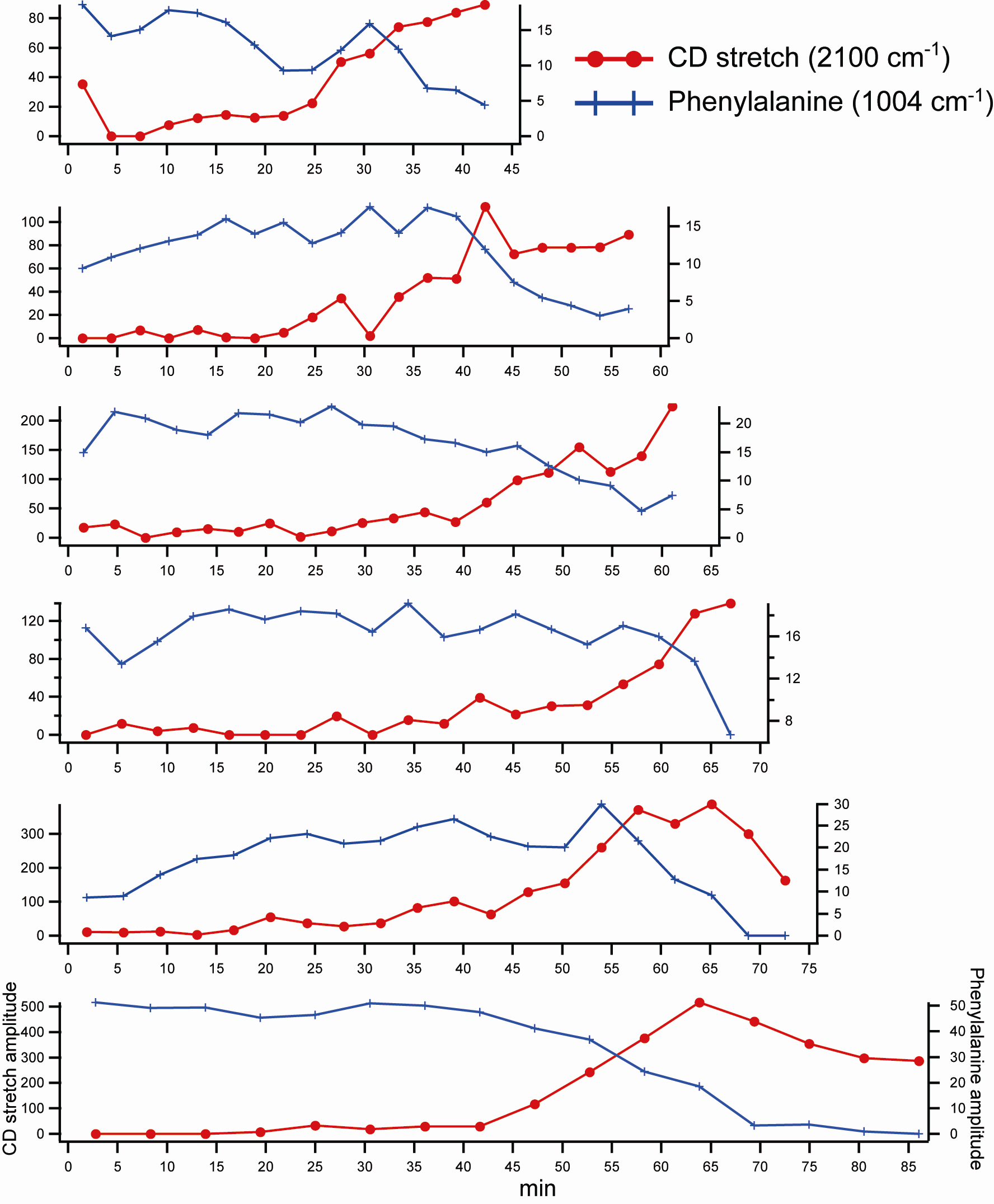


Figure S3 Time-profiles of the Im[χ^(3)^] amplitudes of the 2100 cm^-1^ (CD stretch; red circle, left axis) and 1004 cm^-1^ (phenylalanine; blue cross, right axis) obtained from six CHL cell with the addition of nocodazole.
